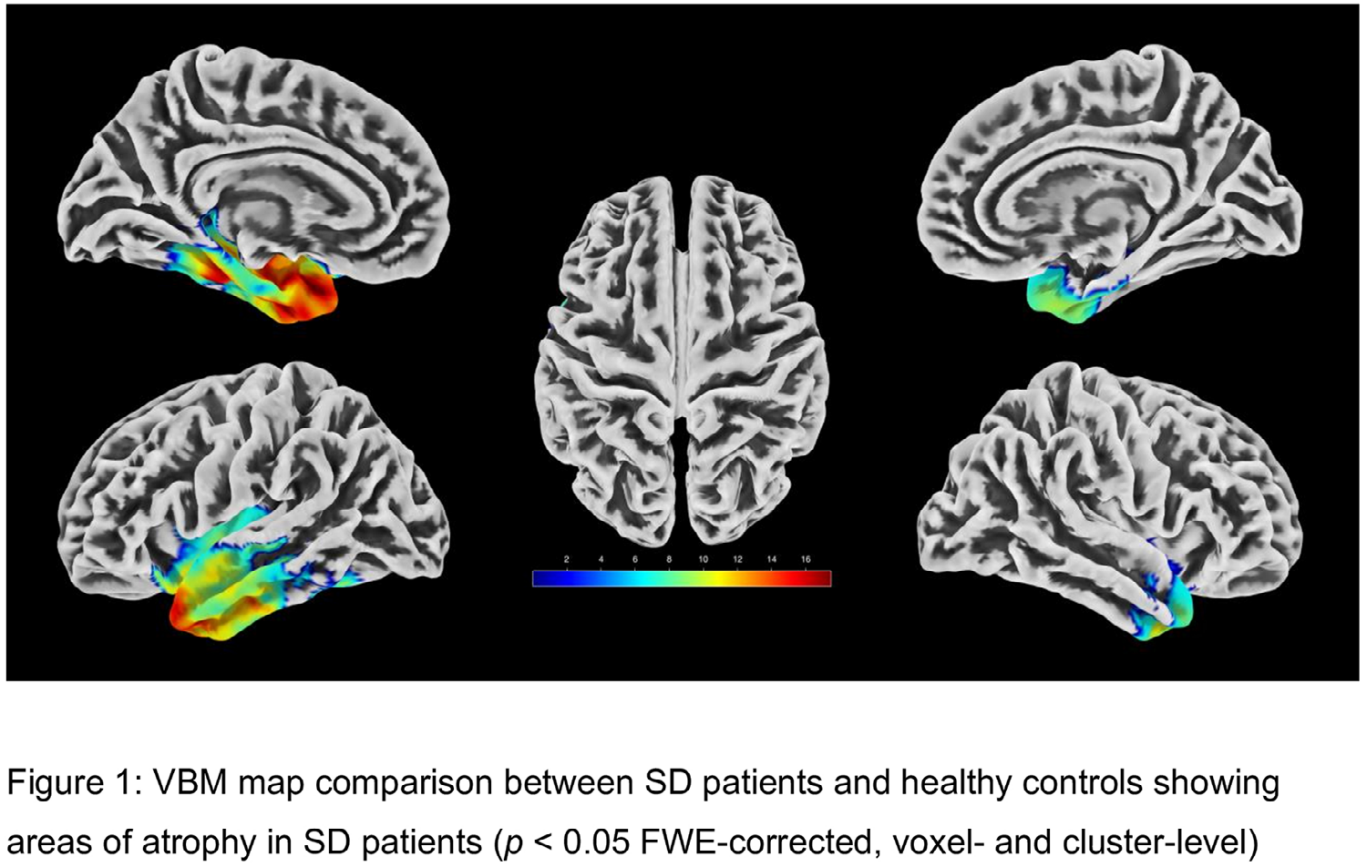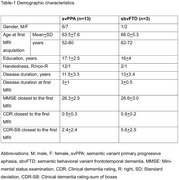# Sequential neuroanatomical pattern of TAR DNA‐binding protein 43 type C propagation using imaging and pathological findings

**DOI:** 10.1002/alz.093527

**Published:** 2025-01-03

**Authors:** Golnaz Yadollahikhales, Ellisa Lang, Diego L. Lorca‐Puls, Maria Luisa Mandelli, Janhavi Pillai, Zachary Miller, Jessica Deleon, Hulya Ulugut, Lea T. Grinberg, Salvatore Spina, Bruce L. Miller, William Seeley, Maria Luisa Gorno Tempini

**Affiliations:** ^1^ Jona Goldrich center for Alzheimer’s and Memory disorders, Department of Neurology, Cedars Sinai Medical Center, Los Angeles, CA USA; ^2^ Memory & Aging Center, Department of Neurology, University of California in San Francisco, San Francisco, CA USA; ^3^ Sección de Neurología, Departamento de Especialidades, Facultad de Medicina, Universidad de Concepción, Concepción, CA Chile

## Abstract

**Background:**

Frontotemporal lobar degeneration (FTLD)‐ TAR DNA‐binding protein 43 (TDP) type C is commonly associated with a clinical diagnosis of semantic dementia (SD). Although anterior temporal lobe (ATL) is one of the primary atrophy centers, it is yet to be defined which other areas are involved in the TDP‐type C pathology early in the disease course.

**Methods:**

We included 16 patients with autopsy‐confirmed FTLD‐TDP type C from the database of the UCSF Memory and Aging Center: 13 patients with semantic variant primary progressive aphasia (svPPA) and predominant left ATL atrophy, and 3 patients with semantic behavioral variant frontotemporal dementia (sbvFTD) and predominant right ATL atrophy. The inclusion criteria required a high‐resolution T1‐weighted MRI scan within four years of disease onset, a Mini‐Mental Status Examination (MMSE) score ≥ 21, clinical dementia rating (CDR) ≤ 1 and CDR‐sum of boxes (SOB) ≤ 9. We employed voxel‐based morphometry (VBM) in SPM12 to identify regions of significant atrophy comparing the SD patients to a group of healthy controls (HC) (N = 29, Mean age 67+6, female = 19). Additionally, we developed a composite neurodegeneration score by summing the neuron loss scores with the mean values of astrogliosis and microvacuolation, as assessed by a neuropathologist.

**Results:**

Voxel‐based morphometry (VBM) analysis comparing SD patients to healthy controls revealed greatest atrophy in the left temporal pole, with subsequent involvement of the left amygdala, entorhinal area, fusiform gyrus, parahippocampal area, and inferior temporal gyrus (ITG). Conversely, the lingual gyrus and inferior occipital area followed by right postcentral, precentral, and angular gyri exhibited the least volume loss. These imaging findings align with autopsy results, indicating that areas with the highest and least volume loss correspond to regions with greater and lower neurodegenerative changes, respectively.

**Conclusions:**

Our results highlighted the potential trajectory of TDP‐type C pathology over the disease course which likely originates in the ATL and extends posteriorly and inferiorly to affect the ITG and parahippocampal regions. The progression pattern suggests that sensorimotor centers and the angular gyrus, as well as the regions associated with primary visual processing, tend to be compromised in the later stages of the disease based on both imaging and neuropathological findings